# Plasma and tumor levels of Linc-pint are diagnostic and prognostic biomarkers for pancreatic cancer

**DOI:** 10.18632/oncotarget.12365

**Published:** 2016-09-30

**Authors:** Le Li, Guang-Quan Zhang, Hua Chen, Zhong-Jie Zhao, Hong-Ze Chen, Huan Liu, Gang Wang, Yue-Hui Jia, Shang-Ha Pan, Rui Kong, Yong-Wei Wang, Bei Sun

**Affiliations:** ^1^ Department of Pancreatic and Biliary Surgery, The First Affiliated Hospital of Harbin Medical University, Harbin, Heilongjiang, China; ^2^ Department of Epidemiology and Biostatistics, School of Public Health, Qiqihar Medical University, Qiqihar, Heilongjiang, China

**Keywords:** pancreatic cancer, prognosis, biomarker, Linc-pint, CA19-9

## Abstract

Long intergenic non-protein coding RNA, p53 induced transcript (Linc-pint) is a long noncoding RNA (lncRNA) that regulates tumor cell viability and proliferation. We used qRT-PCR and RNA FISH analysis to evaluate Linc-pint levels in the plasma and tumor tissues of pancreatic cancer (PCa) patients. Our data demonstrate that Linc-pint expression is lower in plasma samples from PCa patients than from healthy individuals, and indicate that plasma Linc-pint levels are more sensitive than CA19-9 for detecting PCa. Our data also show that Linc-pint levels are lower in PCa tumors than in adjacent tissues, carcinoma of the ampulla of Vater (CAV) and cholangiocarcinoma (CCA), and suggest that Linc-pint could be used for distinguishing the cause of malignant obstructive jaundice. Low plasma Linc-pint levels correlate with tumor recurrence, while low tumor Linc-pint levels correlate with poor prognosis for PCa patients after pancreatectomy. These results thus indicate that low plasma Linc-pint expression could serve as a minimally invasive biomarker for early PCa detection, and that low Linc-pint levels in PCa tumors could be used for predicting patient prognosis.

## INTRODUCTION

Pancreatic cancer (PCa) is one of the most lethal malignancies. In China, incidence and mortality rates of PCa rank ninth and seventh, respectively, among all cancers [1]. Most patients with PCa are asymptomatic until the cancer spreads by metastasis. Therefore, only about 20% of patients have the opportunity to receive curative resection [2]. However, the improvement of surgical resection does not avoid the eventual aggressive tumor micrometastasis and recurrence. The five-year overall survival is less than 5%, combining with postoperative chemotherapy and radiotherapy [3]. To improve the prognosis, accurate biomarkers and molecular targets for PCa are needed to screen patients at high risk and allow for early detection. Serum tumor biomarker, carcinoembryonic antigen (CEA) and carbohydrate antigen 19–9 (CA19-9) have been used to diagnose PCa and monitor recurrence after surgery, but their accuracy and detection limits are low [4–5]. Hence, there is a necessity for novel biomarkers with enhanced sensitivity and specificity to improve PCa-related clinical decisions.

Long noncoding RNAs (lncRNAs), which are more than 200 nt in length and lack functional open reading frames (ORFs), influence different cellular functions in tumorigenesis, including proliferation, metastasis and survival. Previous studies have demonstrated that lncRNAs are detectable in plasma, and their altered expression contributes to cancer development [6–8]. Thus, plasma levels of lncRNAs might serve as potential markers for early PCa detection, tumor status screening, and recurrence monitoring in clinical practice.

Long intergenic non-protein coding RNA, p53 induced transcript (Linc-pint) is ubiquitously expressed in humans and acts as a direct p53 transcriptional target. A previous study has demonstrated that Linc-pint regulates tumor cell viability and proliferation via the induction of cellular apoptosis and DNA damage [9]. Additionally, a multistage, genome-wide association study identified Linc-pint as a new susceptibility candidate gene implicated in PCa development [10]. However, the specific role of Linc-pint in PCa development and progression remains to be explored.

In this study, we have investigated the potential use of Linc-pint as a biomarker for early detection and prognosis in PCa. Most of patients (more than 80%) enrolled in our study were PDACs. Despite the different tumor types and the biological behaviors, our study included pancreatic ductual adenocarcinomas (PDACs), pancreatic cystic adenocarcinomas, pancreatic adenocarcinomas mixed with neuroendocrine carcinomas and pancreatic rare adenocarcinomas. We attempted to investigate the Linc-pint expression levels in different kinds of pancreatic cancers. Our data demonstrate that Linc-pint expression is lower in plasma samples of PCa patients than in healthy individuals, and correlates with tumor recurrence, indicating that the low Linc-pint expression might be used to predict poor prognosis. In addition, Linc-pint expression is lower in PCa tumors compared to adjacent tissues, or to carcinoma of the ampulla of Vater (CAV) and cholangiocarcinoma (CCA). Together, our results provide the first evidence that Linc-pint expression might be used as a minimally invasive biomarker for PCa diagnosis and prognosis.

## RESULTS

### Evaluation of plasma Linc-pint levels from 59 PCa patients

**Figure 1:**
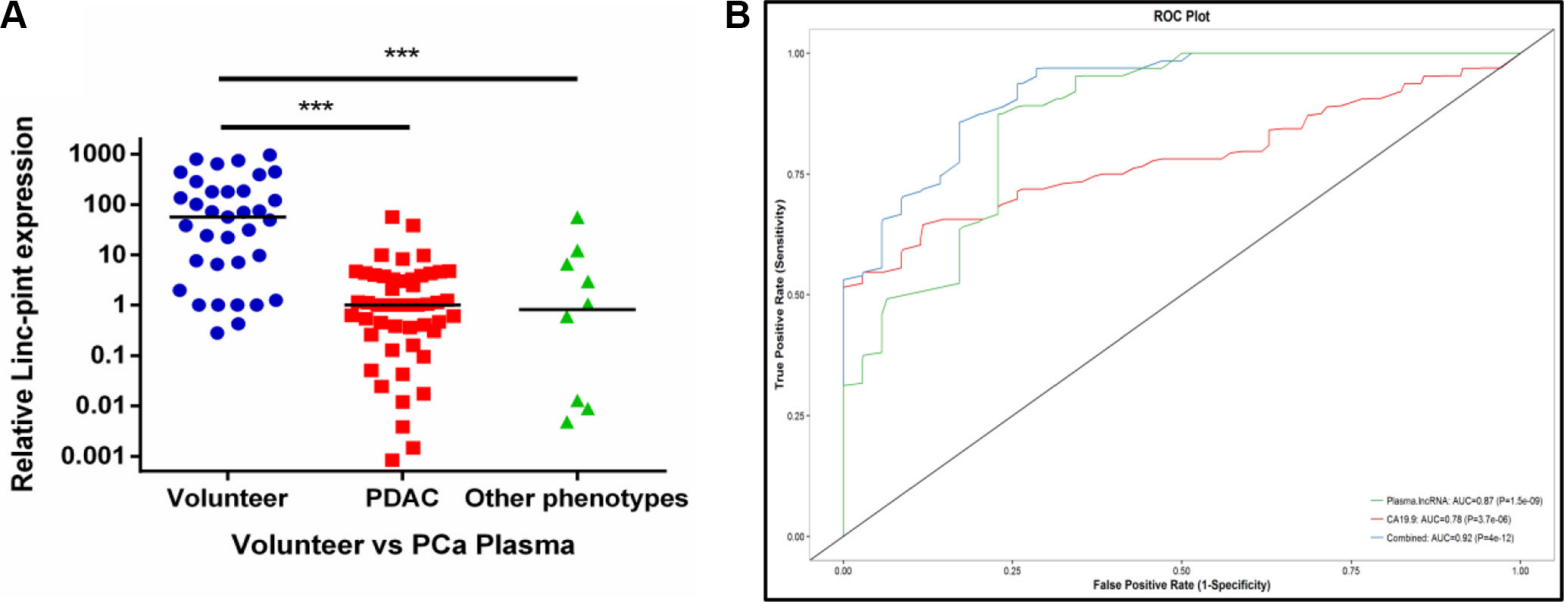
The plasma Linc-pint expression in PCa patients (**A**) Linc-pint levels in plasma from PDAC and other phenotypes PCa patients were significantly lower than in healthy volunteers (*P* < 0.0001, *P* = 0.0005). (**B**) Analysis of receiver-operating characteristic (ROC) curve to detect PCa patients. ROC analysis showed the AUC of plasma Linc-pint was 0.87, the AUC of CA19-9 was 0.78, and the AUC of Linc-pint combined with CA 19-9 was 0.92.

To test the hypothesis that Linc-pint plasma levels could be used for diagnosis of PCa, we measured plasma Linc-pint expression in 59 PCa (49 PDAC and 10 Other phenotypes) patients and 35 healthy volunteers by quantitative real-time reverse transcription-PCR (qRT-PCR) assay Supplementary Table S1 and S2. Plasma Linc-pint levels were significantly lower in PCa patients compared to healthy volunteers (*P* < 0.0001, *P* = 0.0005, Figure 1A). CA19-9 serves as a biomarker for early PCa detection, and its accuracy and detection rates were nearly 71.3% and 73.33%, respectively, as previously described [11]. To detect any cut-off points that could differentiate PCa patients from healthy volunteers, we performed an AUC with Younden index. The area under roc curve (AUC) value of CA19-9 was 0.78, while the values of Linc-pint and Linc-pint combined with CA19-9 were 0.87 and 0.92, respectively (Figure 1B). The receiver operator characteristic (ROC) curves indicated that the sensitivity of plasma Linc-pint (87.5%) for PCa was higher than that of CA19-9 (54.7%), and Linc-pint combined with CA19-9 (85.9%). However, the specificity of plasma Linc-pint (77.1%) was lower than CA19-9 (94.1%), and Linc-pint combined with CA19-9 (82.9%). These data suggest that the plasma Linc-pint levels could be used for predicting PCa, even though the observed higher specificity of CA19-9 for PCa diagnosis should be considered.

### Association between Linc-pint levels and tumor dynamics

**Figure 2:**
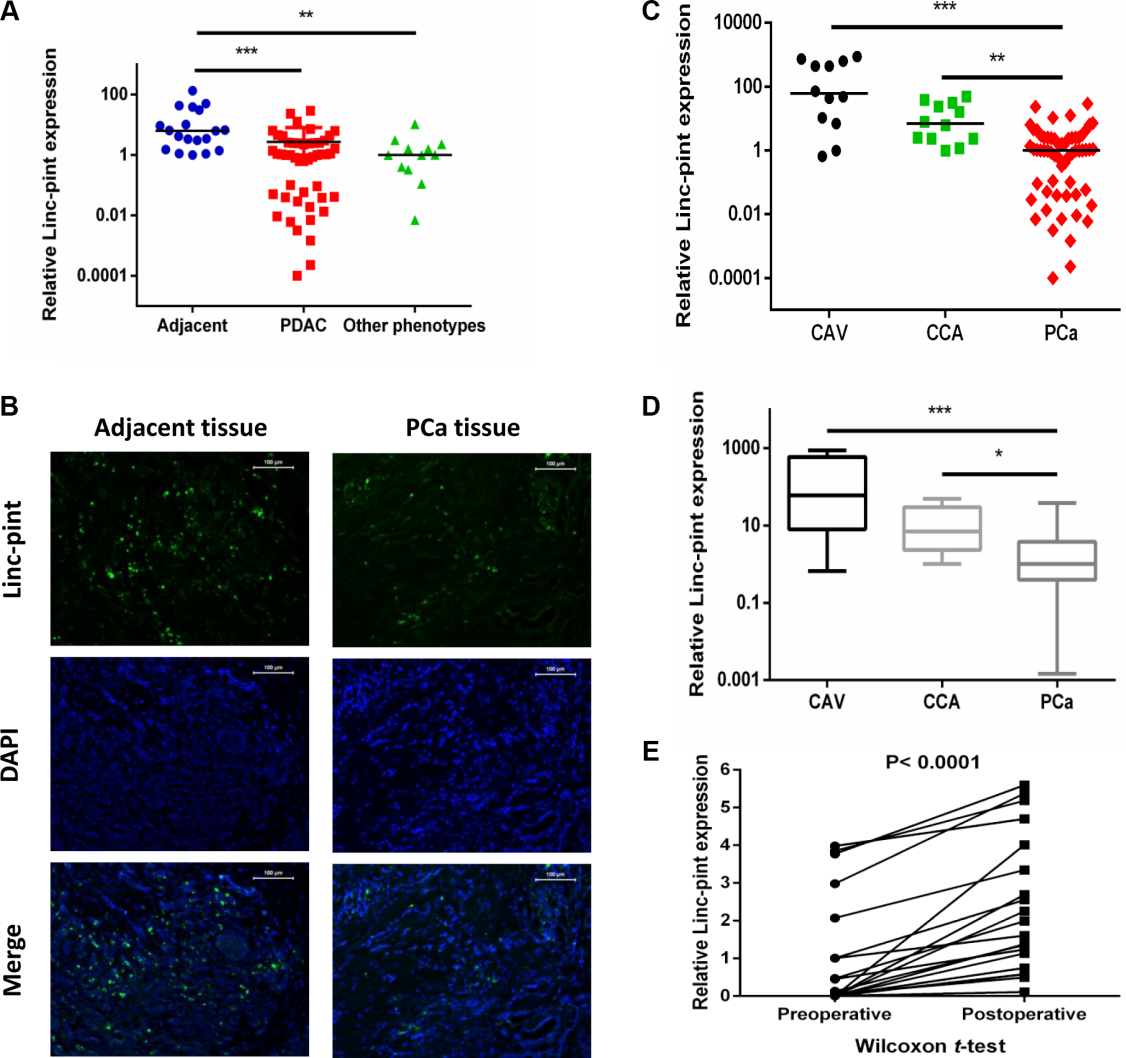
Evaluation of Linc-pint levels in PCa, CAV and CCA patient plasma and tissues The plasma Linc-pint level could reflect tumor dynamics. (**A**) Linc-pint levels were significantly lower in PDAC and other phenotypes PCa tissues than in adjacent tissues ((*P* < 0.0001, *P* = 0.001). (**B**) The RNA FISH assay indicated low Linc-pint levels in PCa tissues compared with adjacent tissues. (**C**) Linc-pint expression was significantly lower in PCa tissues than in CAV and CCA tissues. (**D**) Linc-pint levels were significantly lower in PCa patient plasma than that in CAV and CCA patient plasma. (**E**) Comparison of plasma Linc-pint concentrations between preoperative and postoperative samples from PCa patients. Plasma Linc-pint levels were significantly higher in postoperative samples than in preoperative samples (*P* < 0.001).

To confirm the correlation between Linc-pint levels and PCa, we then evaluated the expression of Linc-pint in PCa tumors and adjacent tissues. The Linc-pint levels were analyzed in 61 PCa (49 PDAC and 12 Other phenotypes) tumors and 19 adjacent tissues (pairs in 19 PCa patients) by qRT-PCR. Linc-pint levels were significantly lower in PCa tissues than in adjacent tissues ((*P* < 0.0001, *P* = 0.001, Figure 2A). This result was confirmed by RNA fluorescence *in situ* hybridization (FISH) assay (six pairs of PCa and adjacent tissues, Figure 2B). The Linc-pint expression of plasma and tumor were compared in PDAC and other tumor phenotypes. There was no significant difference between PDAC and other tumor phenotypes (Supplementary Figure S1A and S1B, *P* = 0.7390, *P* = 0.7100). Additionally, we explored the correlation between plasma Linc-pint expression and tissue Linc-pint expression in the same cohort. The results indicate that there was no significant correlation between plasma and tissue Linc-pint expression (r^2^ = 0.0359, *P* = 0.2073, Supplementary Figure S2). Furthermore, 29 patients were selected from the complete plasma cohort (51 PCa patients) according to the clinical characteristics (malignant obstructive jaundice) and tumor location. Our results suggest that Linc-pint levels are lower in PCa tumors than in CAV and CCA tumors (Figure 2C). In addition, a plasma cohort was used to discuss the different expression levels in PCa, carcinoma of the ampulla of Vater (CAV) and cholangiocarcinoma (CCA) patients. The cohort with 53 patients was composed by these 29 patients, 12 CAV patients and 12 CCA patients and the 29 PCa patients belonged to the cohort with 51 PCa (complete plasma cohort). We then evaluated the plasma Linc-pint levels in the 53 patients who suffered from bile duct obstruction and higher serum bilirubin levels (total bilirubin > 21 umol/L, direct bilirubin > 3.4 umol/L) preoperatively. We observed that plasma Linc-pint levels were lower in PCa patients than in CAV and CCA patients (Figure 2D). The ROC curve analysis was performed to confirm the role of Linc-pint level on identifing malignant obstructive jaundice. We found that the AUC was 0.84 for plasma Linc-pint level and the sensitivity was 89.7% and the specificity was 70.8% at a cut-off value of 5.48 (Supplementary Figure S3). These results indicate that the lower plasma Linc-pint levels could be used for PCa diagnosis in patients with suspected malignant obstructive jaundice. To validate whether Linc-pint plasma levels reflect the tumor dynamics during PCa patient treatment, plasma was collected before and 10 days after surgical resection from 20 patients who underwent curative pancreatectomy. Linc-pint expression was examined in 20 pairs of samples. Our finding show that the Linc-pint levels are elevated postoperatively in the 20 pairs of samples (*P* < 0.001, Figure 2E). These data indicate that Linc-pint levels could reflect tumor dynamics in the course of PCa treatment.

### Correlation between plasma Linc-pint levels and clinicopathological factors in PCa patients

To determine whether the Linc-pint plasma levels correlate with the clinicopathological factors of PCa patients, 51 patients who underwent pancreatectomy were enrolled in this study. We classified the plasma samples into 2 independent cohorts using the median plasma Linc-pint levels. Our results demonstrate that low plasma Linc-pint levels are associated with tumor recurrence (*P* = 0.0428, Table 1). However, no significant association was found between plasma Linc-pint levels, and sex, age, serum CEA and CA19-9 levels, tumor size and location, vascular invasion, lymph node metastasis, tumor stage, T stage, N stage, M stage, and tumor differentiation.

**Table 1:** Relationship between clinicopathological factors and plasma Linc-pint expression in PCa patients

Variables	*N*	Linc-pint		*χ*^2^	*P*-value
		Low	High		
Sex				1.8057	0.1790
Male	35	18	17		
Female	16	5	11		
Age (years)				0.5148	0.4731
< 60	25	10	15		
≥ 60	26	13	13		
CEA				0.1523	0.6964
< 4.3 ug/ml	39	17	22		
≥ 4.3 ug/ml	12	6	6		
CA19-9				0.5436	0.4609
< 37 U/mL	16	6	10		
≥ 37 U/mL	35	17	18		
Tumor size				0.0630	0.8018
< 3 cm	19	9	10		
≥ 3 cm	32	14	18		
Tumor location				1.3030	0.2537
Neck and tail	20	11	9		
Head	31	12	19		
Vascular invasion				0.0309	0.8604
Abssent	48	21	27		
Present	3	2	1		
Lymph node metastasis				0.3755	0.5400
Abssent	29	12	17		
Present	22	11	11		
Tumor stage				0.0093	0.3844
I	8	2	6		
II	20	10	10		
III	21	11	10		
IV	2	0	2		
T stage				4.4532	0.1079
T1	14	3	11		
T2	23	12	11		
T3	14	8	6		
N stage				0.1259	0.7227
N0	28	12	16		
N1	23	11	12		
M stage				0.2965	0.4949
M0	49	23	26		
M1	2	0	2		
Tumor differentiation				0.2792	0.8697
Poor	15	6	9		
Moderate	24	11	13		
Well	12	6	6		
Recurrence				4.100	0.0428*
Absent	38	14	24		
Present	13	9	4		

### Linc-pint levels in PCa tumors could be used for predicting patients' prognosis

**Figure 3:**
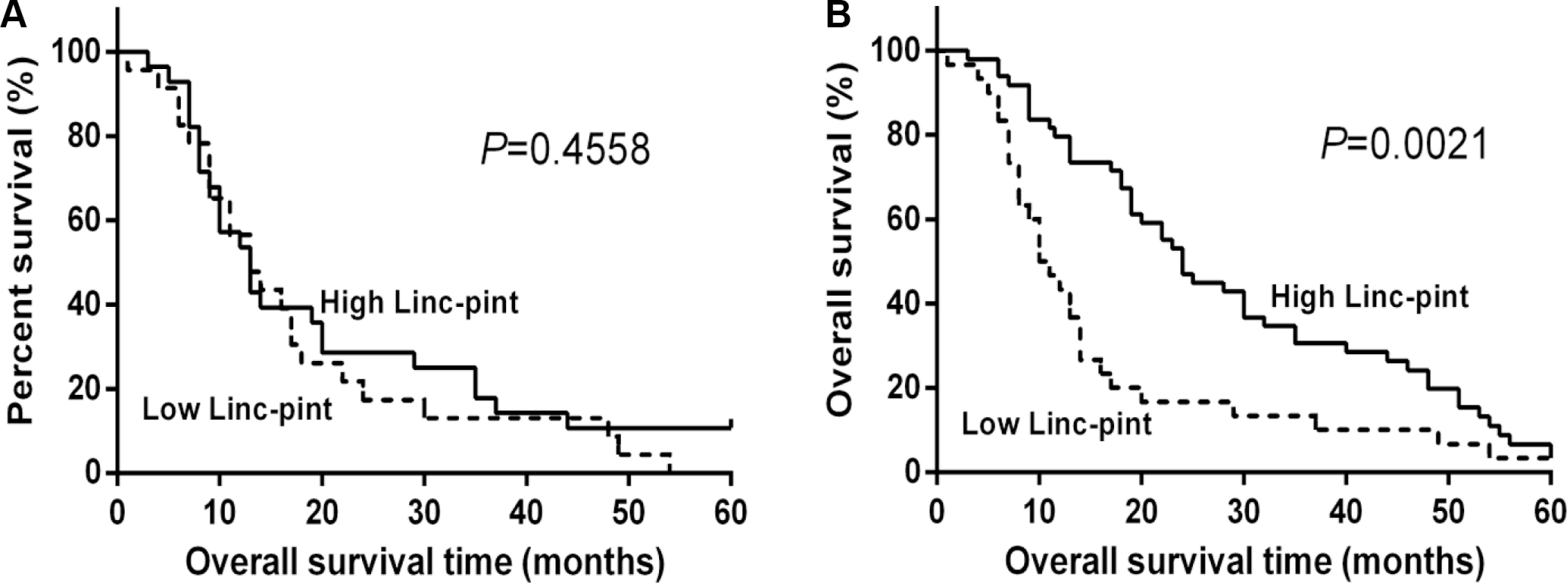
Low tissue Linc-pint levels are associated with a worse prognosis of PCa patients (**A**) Overall survival curves of the high and low plasma Linc-pint level groups of 51 PCa patients who underwent curative pancreatectomy. There is no statistical significance between the two different groups (*P* = 0.4558). (**B**) Overall survival curves of the low and high tissue Linc-pint expression groups of 61 PCa patients who underwent curative resection. The patients with low Linc-pint expression showed significantly poorer long-term prognosis after pancreatectomy (*P* = 0.0021).

Complete follow-up data of PCa patients were available for two cohorts (plasma, *n* = 51; tumors, *n* = 61). The median follow-up time in patients still alive at the time of analysis was 13 months (1–60 months) in both cohorts. As indicated by Kaplan-Meier survival analysis, there was no significant difference in the overall survival between low plasma Linc-pint group and high plasma Linc-pint group (*P* = 0.4558, Figure 3A). However, patients with low Linc-pint levels in tumor tissues had a worse overall survival compared with patients with high Linc-pint tumor levels (*P* = 0.0021, Figure 3B). As shown in Table 2, the above-mentioned prognostic potential of tumor Linc-pint levels was analyzed by univariate Cox proportional hazard regression analysis. Our data revealed that patients with the low Linc-pint tumor levels have shorter overall survival rates (*P* = 0.0095). As expected, serum CA19-9 level (*P* = 0.0059), tumor size (*P* = 0.0040), tumor location (*P* = 0.0430), lymph node metastasis (*P* < 0.0001), tumor stage (*P* < 0.0001), T stage (*P* = 0.0201), N stage (*P* < 0.0001), M stage (*P* = 0.0001) and tumor differentiation (*P* = 0.0015) also served as significant indicators for PCa patient outcome. Furthermore, multivariate Cox proportional hazard regression analysis was performed and revealed that serum CA19-9 level (HR = 2.433, 95% CI = 1.189–4.979, *P* = 0.0149), tumor size (HR = 2.166, 95% CI = 1.154–4.063, *P* = 0.0161), tumor stage (HR = 4.137, 95% CI = 2.103–8.135, *P* < 0.0001), T stage (HR = 4.137, 95% CI = 2.103–8.135, *P* < 0.0001), tumor differentiation (HR = 0.449, 95% CI = 0.203–0.990, *P* = 0.0472) and Linc-pint level (HR = 0.331, 95% CI = 0.177–0.617, *P* = 0.0005) were independent factors predicting the poor prognosis of PCa patients (Table 2).

**Table 2:** Univariate and multivariate analyses in patients with low and high tissue Linc-pint levels

Variables	*P*-value	Multivariate analysis		
		HR	95%CI	*P*-value
Sex (Male, Female)	0.8568			
Age (< 60 years, ≥ 60 years)	0.2113			
CEA (< 5 ug/ml, ≥ 5 ug/ml)	0.2385			
CA19-9 (< 37 U/mL, ≥ 37 U/mL)	0.0059*	2.433	1.189–4.979	0.0149*
Tumor size (< 3 cm, ≥ 3 cm)	0.0040*	2.166	1.154–4.063	0.0161*
Tumor location (Head, Neck and tail)	0.0430*			
Vascular invasion (Yes, No)	0.6131			
Lymph node metastasis (absent, present)	< 0.0001*			
Tumor stage (I/II, III)	< 0.0001*	4.137	2.103–8.135	< 0.0001*
T stage (T1/T2, T3)	0.0201*	2.831	1.407–5.695	0.0035*
N stage (N0, N1)	< 0.0001*			
M stage (M0, M1)	0.0001*			
Tumor differentiation (Poor/Moderate, Well)	0.0015*	0.449	0.200–0.990	0.0472*
Linc-pint (Low, High)	0.0095*	0.331	0.177–0.617	0.0005*

Abbreviations: CI = confidence interval; HR = hazard ratio;

* *P* < 0.05.

## DISCUSSION

Curative pancreatectomy remains the only chance for curing PCa, but only 15%-20% patients present a resectable disease at the time of primary diagnosis [12]. LncRNAs contribute to physiological processes as well as pathological conditions, and play a variety of roles as tumor suppressors, preoperative predictors, and prognostic biomarkers [13–17]. A previous study has demonstrated that upregulation of chromatin-interacting lncRNA, MEG3, inhibits PCa cell proliferation via the activation of p53 [18]. Additionally, lncRNA-NUTF2P3-001 contributes to PCa proliferation and invasion by derepressing the miR-3923/KRAS pathway [19]. Moreover, LOC389641 promotes progression of pancreatic ductal adenocarcinoma and increases cell invasion by regulating E-cadherin with the possible involvement of TNFRSF10A [20]. Insights from such studies have provided evidence that lncRNAs enhance PCa cell proliferation, malignant transformation and metastasis, and that lncRNA regulatory processes are critical players in PCa tumorigenesis. In recent years, plasma lncRNAs have been identified as potential biomarkers of various cancers, including gastric cancer, breast cancer and lung cancer. However, few studies explored the potential use of plasma lncRNAs in PCa detection and prognosis. The only circulating plasma marker, lncRNA HOTTIP, has been used in PCa diagnosis and prognosis [21]. More lncRNAs markers are needed to facilitate early PCa detection.

A genome-wide association study (GWAS) identified multiple susceptibility loci for PCa, which indicated Linc-pint's association with the risk of sporadic PCa. Our present study shows that plasma Linc-pint levels are associated with PCa early detection and tumor dynamics. The plasma Linc-pint levels are significantly lower in PCa patients than in healthy volunteers (*P* < 0.001). Currently, CA19-9 is the best biomarker for detecting PCa, but it is not elevated in almost 25% of PCa patients, and has a 25% false-positive rate [22]. In our study, the AUC value of CA19-9 in PCa detection was 0.78. The sensitivity and specificity of CA19-9 were 54.7% and 94.1%, respectively. Moreover, the AUC value of Linc-pint was 0.87 and the sensitivity and specificity were 87.5% and 77.1%. Combining Linc-pint with CA19-9 could increase the sensitivity of PCa detection in screening programs, but the specificity was lower. However, the specificity of CA19-9 in our study was much higher than in other studies. Thus, the diagnostic accuracy of CA19-9 needs to be further investigated. In addition, we found that the Linc-pint plasma expression was significantly elevated in postoperative samples than in preoperative samples (*P* = 0.003). These data indicate that the plasma Linc-pint levels could serve as a diagnostic and screening marker in PCa patients.

The expression of Linc-pint was much lower in PCa tumors compared to adjacent tissues, indicating that Linc-pint might act as a tumor suppressor in PCa. Furthermore, the Linc-pint plasma levels were lower in patients with pancreatic head cancer than in CAV and CCA patients. In addition, PCa tumors contained lower levels of Linc-pint than CAV and CCA tissues. The AUC for plasma Linc-pint level on identifing malignant obstructive jaundice was 0.84. These data indicate that Linc-pint could be used for distinguishing malignant tumors in the ampullary area, and suggest that Linc-pint could serve as a diagnostic and prognostic marker in PCa patients.

Recent studies have revealed that circulating miRNAs and lncRNAs are associated with tumor dynamics [23–25]. Our results indicate that plasma Linc-pint levels may have an excellent clinical value in predicting tumor recurrence, since the low plasma Linc-pint levels were associated with higher recurrence rates after curative pancreatectomy. LncRNAs have been identified as potential prognostic biomarkers in PCa patients [26–28]. In a previous study, we confirmed that MALAT1 was an independent predictor of poor prognosis in PDAC patients, and MALAT1 upregulation promoted PCa proliferation and metastasis via the stimulation of autophagy [29]. This study demonstrates that low Linc-pint levels in PCa tissues is an independent poor prognosis factor for PCa patients, whereas low plasma Linc-pint levels are not an effective prognosis indicator. Taken together, our data show that the plasma Linc-pint levels could be used for monitoring and predicting tumor recurrence, and tissue Linc-pint levels could be used for predicting patient prognosis.

To our knowledge, this is the first study to consider the use of plasma and tissue levels of Linc-pint in cancer diagnosis and prognosis. Although the present data were limited by the small sample size, our study indicates that the Linc-pint levels could serve as a useful and minimally invasive strategy for diagnosing PCa and predicting patient prognosis. In future, a large-scale prospective study should be performed to validate the accuracy and effectiveness of Linc-pint as a representative biomarker for PCa.

## MATERIALS AND METHODS

### Patients and samples

This study was approved by the Research Ethics Committee of The First Affiliated Hospital of Harbin Medical University, and each patient provided written informed consent. Blood and tissue samples were collected from patients who underwent primary tumor resection from May 2008 to January 2011 in the Department of Pancreatic and Biliary Surgery of The First Affiliated Hospital of Harbin Medical University. No patients received preoperative chemotherapy or radiotherapy. All these volunteers were recruited from the Center of Health Examination in The First Affiliated Hospital of Harbin Medicial University. Diagnostic tests were performed to exclude hepatobiliary and pancreatic diseases and other benign and malignant tumors. Each patient provided written informed consent. The detailed clinical characteristics of these individuals were listed in Supplementary Table S3. We used plasma samples obtained from PCa patients (*n* = 59), CAV patients (*n* = 12), CCA patients (*n* = 12) and healthy volunteers (*n* = 35). In addition, a total of 61 tumor tissues and 19 adjacent tissues from PCa patients, as well as 12 CAV tissues and 12 CCA tissues were used for the qRT-PCR assay. Peripheral blood (5ml) was obtained from each patient preoperatively, and blood was obtained from 20 PCa patients 10 days after curative pancreatectomy. Blood was collected from patients and healthy individuals in sodium heparin tubes (BD Vacutainer, Becton, Dickinson and Company, Franklin Lakes, NJ, USA) and immediately subjected to the three-spin protocol (1,500 r.p.m. for30 min, 3,000 r.p.m. for 5 min, and 4,500 r.p.m. for 5 min) to prevent contamination by cellular nucleic acids. Plasma was transferred to 1.5-ml tubes and stored at −80°C for further processing. The resected specimens were fixed in formalin and embedded in paraffin for pathological diagnosis and FISH assay. Portions of these specimens were immediately snap frozen in liquid nitrogen until RNA extraction. All patients had a histological diagnosis and were followed up every 3 months. Clinicopathological factors of PCa were classified using the TNM system of classification. All PCa patient data, including age, gender, clinical manifestation, tumor biomarker, tumor size and location, lymph node metastasis, vascular invasion, clinical stage, histological grade and recurrence were obtained from the clinical and pathological records. Overall survival was calculated from the date of surgery to the date of death. Patients who died of other diseases not related to PCa were excluded from our study.

### RNA isolation and quantitative real-time reverse transcription-PCR

RNA isolation, reverse transcription and qRT-PCR were performed as described previously [30–31]. Total serum and tissue RNA was extracted by the RNA Isolation Kit (AXYGEN, Union City, CA, USA) according to the manufacturer's instructions. The cDNA was synthesized from total RNA using the TOYOBO Kit (Osaka, Japan). qRT-PCR was performed on theApplied Biosystem 7500 with SYBR Green (Roche, USA). Relative Linc-pint expression was quantified using the 2^−ΔΔCT^ method after normalization for the expression of the control, and the expression of GAPDH served as the endogenous control. The Linc-pint and GAPDH primer sequenceswere as follows: Linc-pint forward (5′ to 3′): CGTGGGAGCCCCTTTAAGTT, reverse (5′ to 3′):GGGAGGTGGCGTAGTTTCTC; GAPDH forward (5′ to 3′): CCTCTGACTTCAACAGCGACAC, reverse (5′ to 3′): TGGTCCAGGGGTCTTACTCC.

### RNA fluorescence *in situ* hybridization

Formalin-fixed paraffin-embedded sections (4μm) were baked at 45°C overnight. RNA *in situ* hybridization was performed using a Linc-pint probe (cctgggatgaatcgggaggagcggtggagactccggagacaggtgccgcgctggtctgggctgccggctaaaagttgtcctccgcgcggtgggcggtggggtccccggccagggccaaggaccccggctccctgccccccggcgtcgcccacttgtcacgccaggctgctgcgtgcactcggctgcaggggaggtggcgtagtttctcttcctcccacctcttctcactcacttgtttttgtagccgtgg). Tissues were deparaffinized by immersing in fresh xylene twice and then digested in proteinase K. Slides were immersed in wash buffer and treated with pre-cooled ethanol. Slides were then incubated with FITC-labelled target probes overnight, and counterstained with 4′6-diamino-2-phenylindole (DAPI, Beyotime, Nanjing, China). Finally, the slides were viewed using a laser scanning confocal microscope (40×, Olympus, Japan).

### Statistical analysis

All data were analyzed using SAS (version 9.3, SAS Institute, Cary, NC, USA). Data were expressed as the mean ± s.d. Differences between two groups were estimated by the χ^2^ test, Student's *t-test*, Mann-Whitney *U-test*, and Wilcoxon test. Multiple comparisons between more than two groups were assessed by the Kruskal-Wallis test. The relationships between plasma and tumor Linc-pint levels were analyzed by Pearson's analysis. ROC curve analysis and the AUC statistic were used to measure the accuracy of plasma Linc-pint, CA19-9 and Linc-pint combined with CA19-9 in identifying PCa. Univariate and multivariate analyses were used to establish the correlation between plasma and tissue Linc-pint expression with clinical and pathological characteristics. The Kaplan-Meier method and log-rank test were applied to compare the survival of different groups. Multivariate Coxproportional hazard regression analysis was performed to assess the hazard ratios of overall survival according to different plasma and tissue Linc-pint levels. All differences were considered statistically significant at the level of *P* < 0.05.

## SUPPLEMENTARY MATERIALS FIGURES AND TABLES






