# Supplementary material for: Plasma and tumor levels of Linc-pint are diagnostic and prognostic biomarkers for pancreatic cancer
Source: Oncotarget. 2016 Sep 30;7(44):71773–81. doi: 10.18632/oncotarget.12365 (PMC5342121; doi:10.18632/oncotarget.12365)
Supplement: Supplementary file 1 [file oncotarget-07-71773-s001.pdf]

Plasma and tumor levels of Linc-pint are diagnostic and prognostic biomarkers for pancreatic cancer

Supplementary Materials

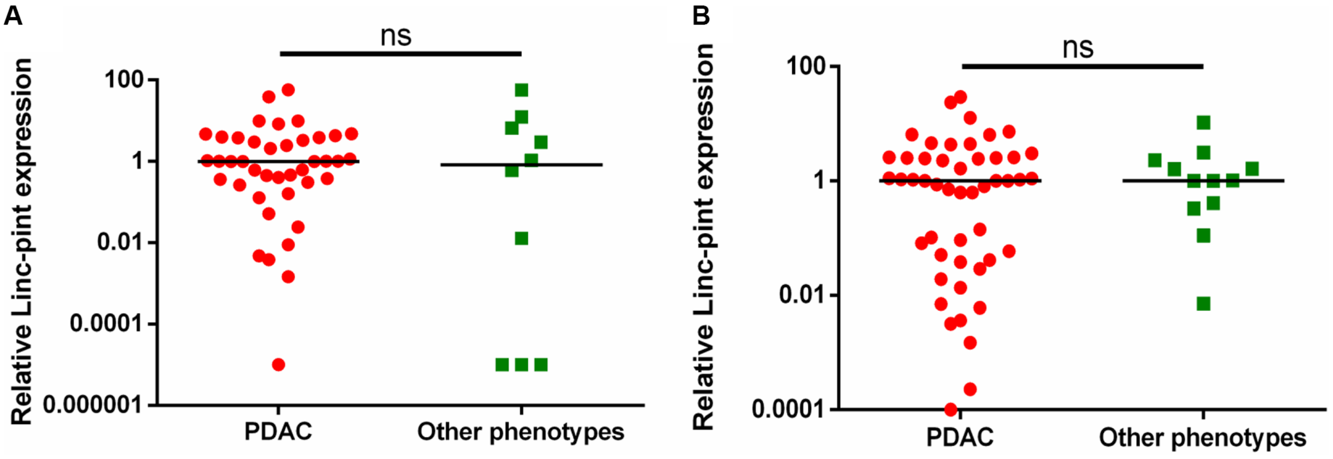

Supplementary Figure S1: (**A**) Relative plasma Linc-pint expression of PDAC and other tumor phenotypes (P = 0.7390). (**B**) Relative tissue Linc-pint expression of PDAC and other tumor phenotypes (P = 0.7100).

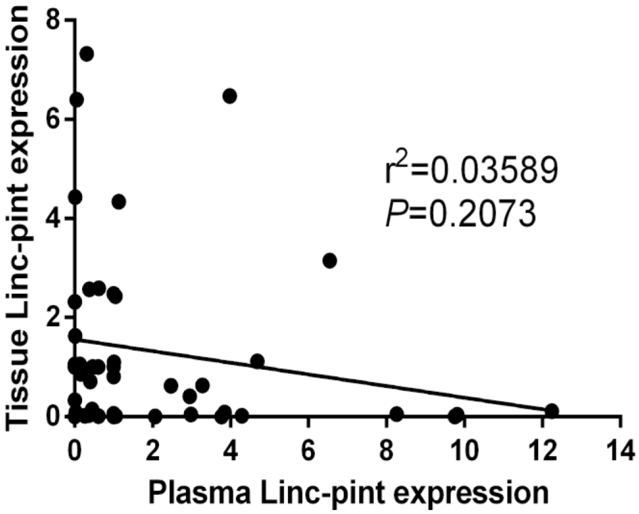

Supplementary Figure S2: The correlation between the plasma Linc-pint expression and the tissue Linc-pint expression (r^2 = 0.0359, P = 0.2073).

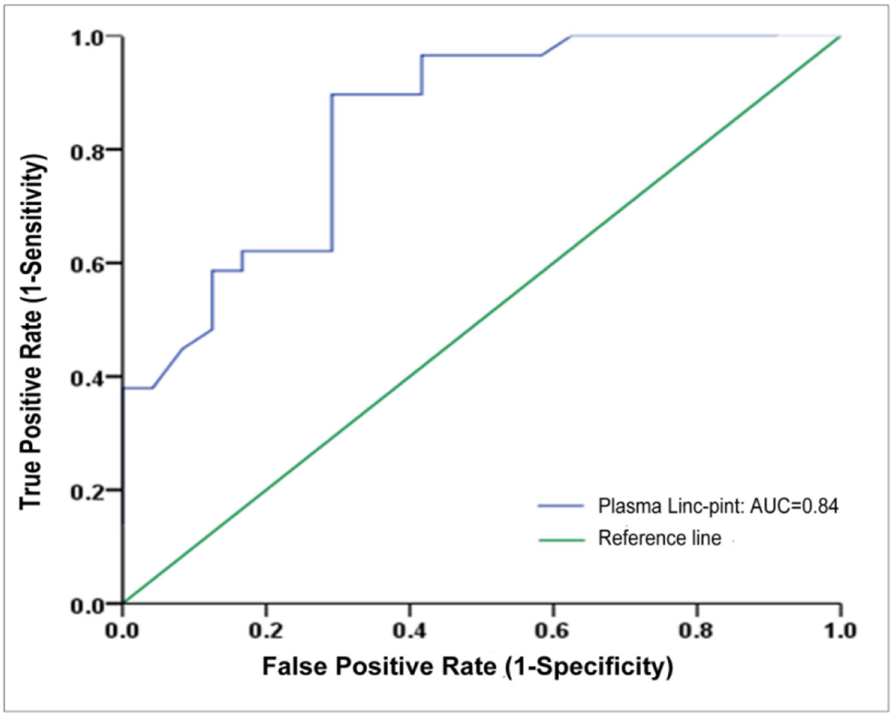

Supplementary Figure S3: Analysis of ROC curve to confirm the role of plasma Linc-pint expression on identifing malignant obstructive jaundice. The AUC of plasma Linc-pint for PCa was 0.84.

Supplementary Table S1: Plasma Linc-pint data. See Supplementary_Table_S1

Supplementary Table S2: Tissue Linc-pint data. See Supplementary_Table_S2

Supplementary Table S3: Healthy volunteers

Number	PLASMA Linc-pint expression	Sex	Age	Ethnicity
1	1.972947	Male	36	Asian
2	31.08319	Male	51	Asian
3	48.96848	Male	67	Asian
4	9.677235	Female	46	Asian
5	7.042507	Female	49	Asian
6	6.508214	Male	38	Asian
7	72.17416	Female	55	Asian
8	101.1327	Male	28	Asian
9	135.2709	Male	44	Asian
10	74.48647	Male	41	Asian
11	56.70361	Male	56	Asian
12	38.31213	Female	59	Asian
13	70.33121	Male	62	Asian
14	0.4263959	Male	66	Asian
15	1.241822	Female	64	Asian
16	440.833	Male	43	Asian
17	799.62	Male	53	Asian
18	745.192	Female	34	Asian
19	444.0872	Female	57	Asian
20	969.4844	Male	51	Asian
21	1	Female	62	Asian
22	7.680307	Female	41	Asian
23	1	Female	56	Asian
24	393.0324	Female	70	Asian
25	120.7281	Female	38	Asian
26	187.048	Female	35	Asian
27	0.2804756	Male	44	Asian
28	178.311	Male	47	Asian
29	639.9928	Male	35	Asian
30	1	Female	31	Asian
31	287.7191	Male	50	Asian
32	24.08066	Female	55	Asian
33	1	Male	70	Asian
34	177.9698	Female	62	Asian
35	22.21584	Male	40	Asian
